# Association between environmental mercury exposure and allergic disorders in Korean children: Korean National Environmental Health Survey (KoNEHS) cycles 3–4 (2015–2020)

**DOI:** 10.1038/s41598-024-51811-3

**Published:** 2024-01-17

**Authors:** Ji-youn Lee, Yun-Hee Choi, Hyeon-il Choi, Kyong Whan Moon

**Affiliations:** 1 School of Health and Environmental Science, Korea University, Anam-ro 145, Seongbuk-gu, Seoul, 02841 South Korea; 2 Department of Ophthalmology, Korea University, Anam-ro 145, Seongbuk-gu, Seoul, 02841 South Korea; 3 Department of Infection Biology, College of Medicine, Chungnam National University, Munhwa-ro 266, Jung-gu, Daejeon, 35015 South Korea; 4 BK21 FOUR R&E Center for Learning Health System, Korea University, Anam-ro 145, Seongbuk-gu, Seoul, 02841 South Korea

**Keywords:** Public health, Risk factors

## Abstract

Although previous studies have suggested potential adverse effects of mercury on a child’s immune system, the associations have been inconsistent. We aimed to determine the association between urinary mercury levels and allergic diseases in Korean children with high mercury exposure. Data from 853 and 710 children aged 6–11 years in the Korean National Environmental Health Survey (KoNEHS) cycle 3 (2015–2017) and cycle 4 (2018–2020) were analyzed. We examined the association between mercury exposure and the prevalence of atopic dermatitis (AD), asthma, allergic rhinitis (AR), and allergic multimorbidity. After adjusting for all covariates, the urinary mercury level was positively associated with AD in the 2015–2017 study (OR = 1.34, 95% CI = 1.01, 1.79) and AR in 2018–2020 study (OR = 1.46, 95% CI = 1.01, 2.10). Pooled effects showed OR of 1.34 (95% CI = 1.01, 1.79) for AD and 1.47 (95% CI = 1.01, 2.12) for allergic multimorbidity. The association with allergic multimorbidity was greater in boys (OR = 1.88, 95% CI = 1.01, 3.49) than in girls (OR = 1.25, 95% CI = 0.73, 2.14). These results suggest that environmental mercury exposure may exacerbate symptoms of atopic dermatitis and allergic multimorbidity in children.

## Introduction

In modern society, changes in the environment, such as industrialized lifestyle habits and air pollution, cause disruptions to the immune system by an overactive immune response to external antigens, leading to an increase in allergic diseases^1^. Allergic diseases include atopic dermatitis, asthma, and allergic rhinitis. As one of the most common chronic diseases among children and adolescents worldwide, allergic diseases mainly appear in childhood and have different patterns depending on age^2,3^. Allergic diseases have negative effects on both the physical and psychological quality of life of patients and are a public health problem that increases the economic burden on society. Therefore, it is important to identify and manage risk factors for allergic diseases.

Genetic factors, diet, exercise, environmental pollution, climate change, and exposure to chemicals have been identified as risk factors for allergic diseases^4–6^, with heavy metals being a major public health issue as they are not easily broken down and can accumulate in the body, causing harm. Among heavy metals, mercury is mainly encountered daily through the consumption of grains, fish, and dental amalgams^7^. Mercury exposure has been reported to have negative effects on the immune system by disrupting the pathways of various signaling molecules, including cytokines, as demonstrated in experimental studies^8,9^. These effects may also be observed in humans in vivo; epidemiological studies targeting children are needed as heavy metals may have a greater impact on vulnerable groups such as children^10^.

Several previous studies have reported that mercury is a risk factor for allergic diseases^7,11,12^. However, in other studies, a significant association was not reported, and the relationship between mercury and allergic diseases remains inconclusive^13,14^. Moreover, studies on children were generally conducted using cord blood^12,15^, and these studies focused on specific diseases despite the coexistence of multiple symptoms in allergic diseases^12,15^. In Korea, where the consumption of grains and seafood is high, the mercury concentration in the body is higher than that in Western countries^16^, and its health implication is expected to be considerable. Therefore, epidemiological studies targeting Korean children are needed.

In this study, we aimed to investigate the associations of urinary mercury concentrations with atopic dermatitis, asthma, allergic rhinitis, and multimorbidity among school-aged children using data from a general representative population of children in Korea.

## Materials and methods

### Study population

This study was conducted using data from the Korean National Environmental Health Survey (KoNEHS). KoNEHS is a legal survey conducted every three years by the Korean National Institute of Environmental Research (NIER) to identify the exposure levels and trends of environmentally harmful substances in the Korean general population^17^. Participants were selected from elementary school students aged 6–11 years, who were prone to exposure to traffic-related air pollution and various indoor allergens in the school environment^18^. Data from the 3rd and 4th surveys were used for the current study. In the 3rd survey (2015–2017), 887 participants were recruited, and in the 4th survey (2018–2020), 736 participants were recruited. Among those, 34 and 26 participants without information on covariates such as sex, age, average monthly household income, and urine cotinine level were excluded from this study. Therefore, 853 and 710 study participants were finally included in the analysis for the 3rd and 4th surveys, respectively.

The Institutional Review Board (IRB) of the National Institute of Environmental Research (NIER), Korea, approved the KoNEHS (IRB No. NIER-2016-BR-003-01, NIER-2016-BR-003-03, NIER-2018-BR-003-02). All methods in this study were conducted in accordance with the relevant guidelines and regulations and every participant submitted written informed consent.

### Measurement of urinary mercury levels

In the KoNEHS, the NIER conducted environmental pollutant analyses. For children, only urine, not blood, samples were collected, considering sample stability. Urine samples were collected the day before or on the morning of the day of the survey by the guardian of each participant, and the collected urinary samples were kept frozen at − 20 °C until analysis. The mercury concentration in urine was analyzed at a wavelength of 253.7 nm using a gold amalgamation mercury analyzer.

Further details of analysis procedures and quality control are described in the protocol of NIER^17^. In addition, public confidence in the analysis data was secured by participating in the G-EQUAS (The German External Quality Assessment Scheme for Analysis in Biological Materials) twice a year. The reported detection limits for this method were 0.1 µg/L and 0.04 µg/L for the 3rd and 4th surveys, respectively, and concentrations below the detection limit were replaced with values obtained by dividing the detection limit by the square root of 2 (0.07 and 0.028 µg/L, respectively).

### Allergic outcomes

Information on current symptoms of atopic dermatitis, asthma, and allergic rhinitis was collected through a structured questionnaire. Since the participants were children, the guardians of the research participants completed the questionnaire, and trained surveyors checked the questionnaire for the guardians. To determine the current presence of allergies, participants who had received a physician's diagnosis were asked the following two questions: "Do you currently have symptoms of atopic dermatitis/asthma/allergic rhinitis?" and "Are you currently taking medication for atopic dermatitis/asthma/allergic rhinitis?" Individuals who answered "yes" to either of these two questions were considered to have current allergic diseases. As allergic disorders tend to coexist with other symptoms^19^, the presence of multimorbidity was noted if a participant had two or more of the three diseases of atopic dermatitis, asthma, and allergic rhinitis at the same time^20^.

### Covariates

To control for potential confounding factors, the covariates included sex, age, parental education level, monthly household income, body mass index (BMI), urinary cotinine level, and urinary creatinine concentration. All data were collected through KoNEHS surveys, physical examinations, and experimental measurements. Parental education level was classified into three stages (college or associated degree, university, and graduate school) according to the highest educational qualification level of the mother or father; monthly household income was classified into four stages (low, lower-middle, upper-middle, and high). BMI was calculated by dividing the weight (kg) by the square of the height (m^2^). The urinary cotinine level was used as a biomarker for smoking exposure^21^, and the urinary creatinine concentration was used to adjust for the urinary mercury concentration.

### Statistical analysis

As the KoNEHS surveyed based on a two-stage proportionally stratified sampling design, we conducted the statistical analysis using the complex sampling design, which considered sampling unit, strata, and sampling weights^17^. Because KoNEHS does not provide integrated weights for each cycle or data to calculate them, the data from the 3rd and 4th cycles were analyzed separately. Due to the left-skewed distribution, urinary mercury, urinary cotinine, and urinary creatinine concentrations were natural log-transformed before the analysis. Age, urinary creatinine concentration, urinary cotinine concentration, and BMI were used as continuous variables, whereas sex, parental education level, and monthly household income were used as categorical variables.

To compare the average mercury levels among demographic groups, independent *t*-tests and Wald *F* tests were used. Logistic regression analysis was used to confirm the association between mercury concentration and allergic disorders, and odds ratios (ORs) and 95% confidence intervals (CIs) were calculated. Furthermore, multinomial logistic regression analysis was used to confirm the relationship between urinary mercury concentration and the multimorbidity of each disease. The multimorbidity of allergic disorders was grouped into "none," "one symptom," and "two or three symptoms," depending on the individual's symptom count. The regression model was adjusted for sex, age, parental education level, monthly household income, BMI, urinary cotinine concentration, and urinary creatinine concentration. A stratified analysis was conducted to compare the magnitude of the association by sex. To calculate the overall effect size in the 3rd and 4th cycles, fixed-effect models were calculated using the Mantel–Haenzel method in the R “meta” package.

Statistical analyses were performed using SPSS 26.0 and R 4.2.2, and statistical significance was defined as *p* < 0.05.

## Results

Table 1 shows the demographic and clinical characteristics of the study participants of the 3rd and 4th cycles. The average age by cycle was 8.50 years and 8.53 years, respectively, with about half of the participants being male in both periods. The prevalence rates of allergic disorders in the 3rd and 4th cycles were 9.3% and 8.9% for atopic dermatitis, 0.8% and 0.8% for asthma, and 33.1% and 32.1% for allergic rhinitis, respectively, with no significant difference by cycle.

**Table 1:** Demographic and clinical characteristics of study participants.

Characteristics	Cycle 3	Cycle 4
	(n = 853)	(n = 710)
Age (AM ± SD)	8.50 ± 1.69	8.53 ± 1.68
Sex (n, %)		
Male	437 (51.2)	339 (47.7)
Female	416 (48.8)	371 (52.3)
Parental education (n, %)		
College or associated degree	387 (45.4)	310 (43.7)
University	344 (40.3)	296 (41.7)
Graduate school	122 (14.3)	104 (14.6)
Household income (million Won/month) (n, %)		
Low (< 3)	170 (19.9)	119 (16.8)
Mid-low (3–4.99)	318 (37.3)	279 (39.3)
Mid-high (5–6.99)	237 (27.8)	206 (29.0)
High (≥ 7)	128 (15.0)	106 (14.9)
BMI (kg/m^2^)^1^ (n, %)		
Underweight/normal	726 (85.1)	604 (85.1)
Overweight	85 (10.0)	72 (10.1)
Obese	42 (4.9)	34 (4.8)
Urinary cotinine level (µg/L) (GM ± GSD)	1.16 ± 2.99	1.67 ± 2.89
Urinary creatinine level (g/L) (GM ± GSD)	1.07 ± 1.66	1.14 ± 1.65
Allergic disorders (n, %)		
Atopic dermatitis	79 (9.3)	63 (8.9)
Asthma	7 (0.8)	6 (0.8)
Allergic rhinitis	282 (33.1)	228 (32.1)

*AM* arithmetic mean, *BMI* body mass index, *GM* geometric mean, *GSD* geometric standard deviation, *SD* standard deviation.

^1^BMI was categorized into three groups: underweight or normal, which was defined as < 85th percentile; overweight, which was 85th–95th percentile; and obese, which was ≥ 95th percentile^22^.

**Figure 1:**
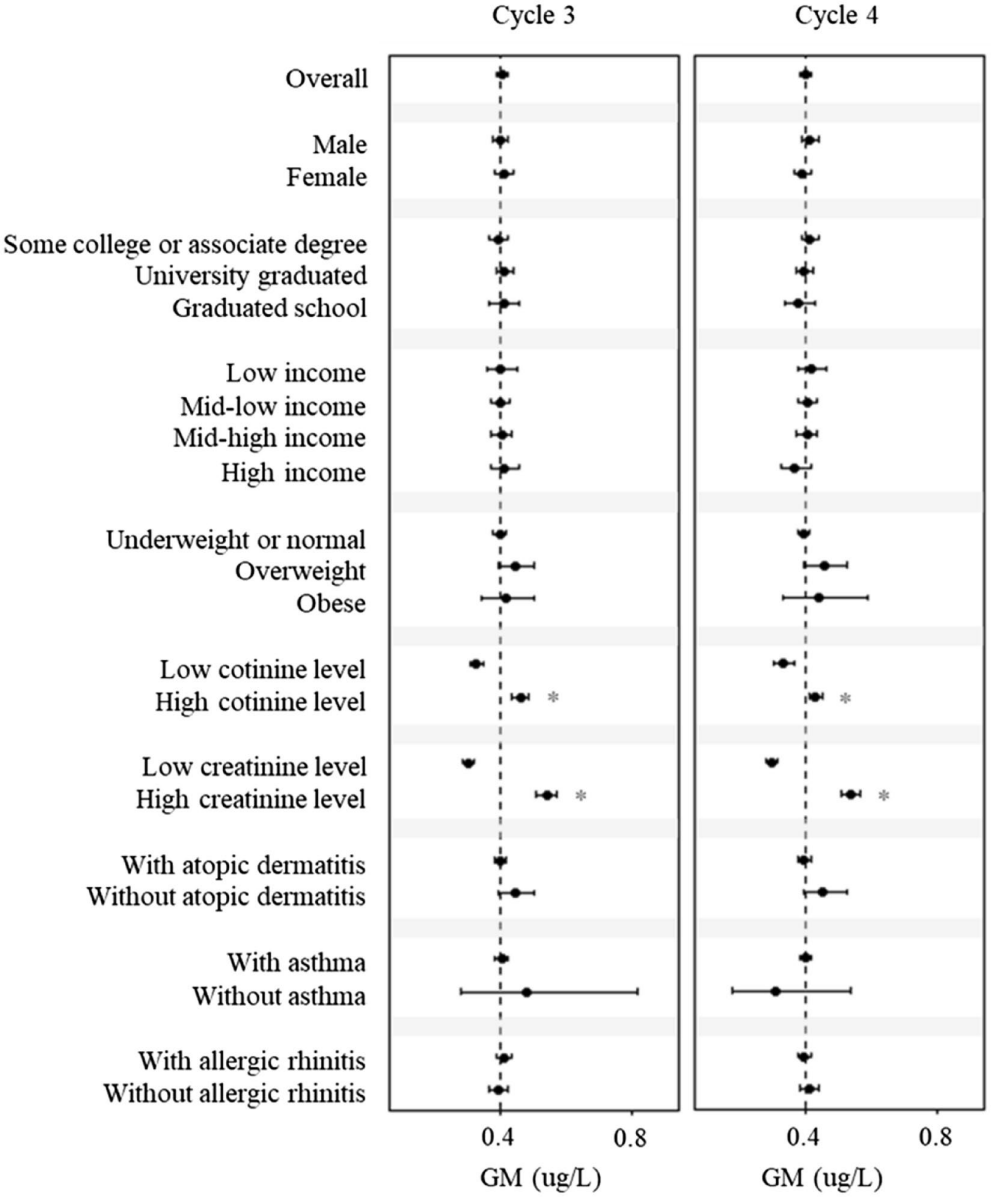
The geometric means (GMs) and 95% confidence intervals of urinary mercury levels (µg/L) by participant’s characteristics. Urinary cotinine levels were classified as low (< 0.1 µg/L) or high (≥ 0.1 µg/L) levels^23^. Urinary creatinine levels were classified based on the median. Red asterisks (*) indicate significant differences between the groups (*p* < 0.05).

Figure 1 shows the urinary mercury levels according to the characteristics of the study participants. The geometric mean of the total urinary mercury concentration was 0.401 µg/L in the 3rd cycle and 0.399 µg/L in the 4th cycle, and there was no significant difference by sex (male vs. female: 0.40 µg/L vs. 0.41 µg/L in the 3rd cycle, 0.41 µg/L vs. 0.39 µg/L in the 4th cycle). In both cycles, mercury concentrations were significantly higher when urinary cotinine and urinary creatinine concentrations were higher (*p* < 0.05). In addition, participants with atopic dermatitis symptoms had higher mercury concentrations than those without atopic dermatitis. There were directional differences in asthma and allergic rhinitis according to the cycle, but they were insignificant.

**Figure 2:**
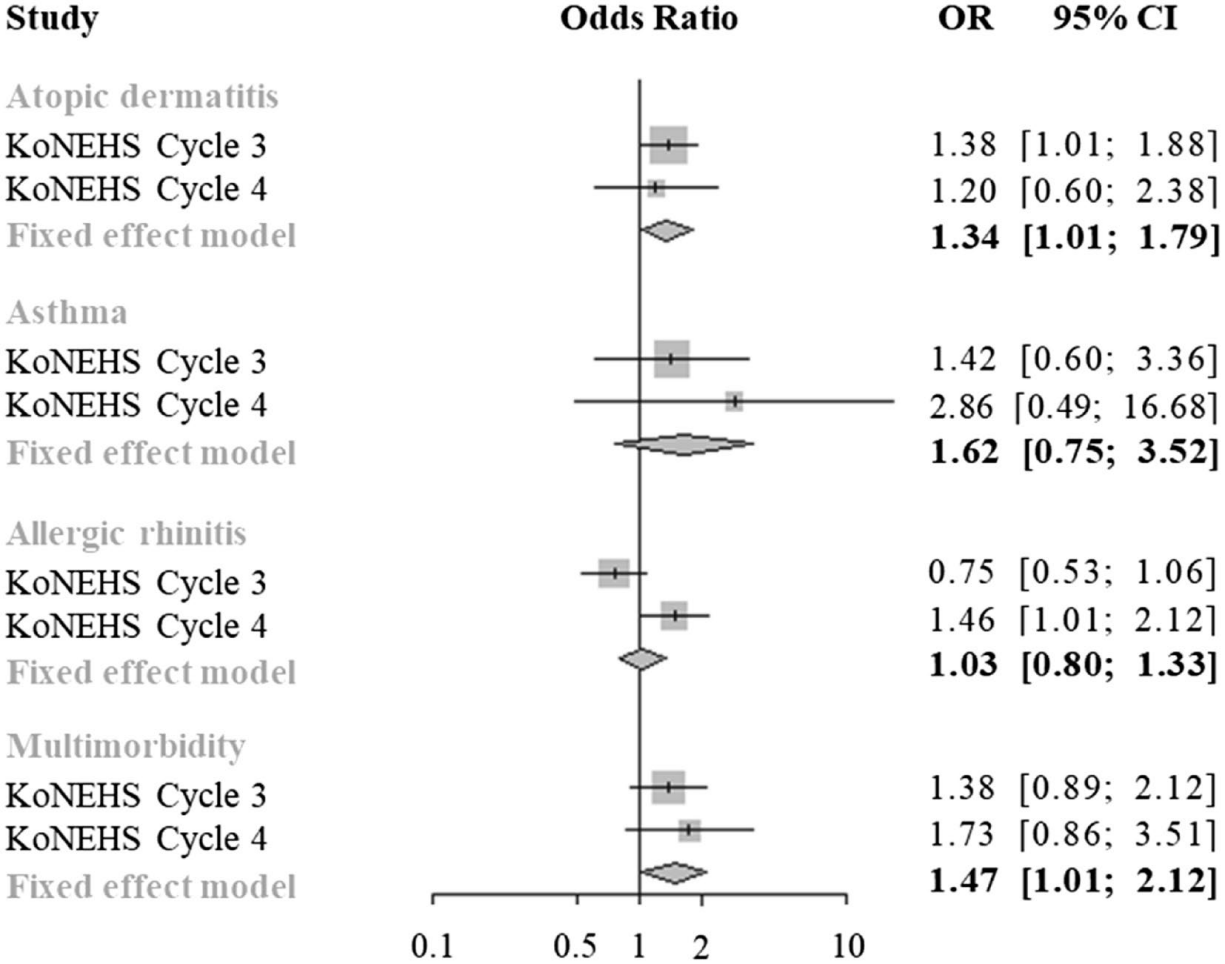
Pooled effects with data from the Korean National Environmental Health Survey (KoNEHS) cycle 3 and cycle 4. All models were adjusted for urinary creatinine level, age, sex, urinary cotinine level, household income, parental education, and body mass index. *CI* confidence interval, *OR* odds ratio.

Table 2 shows the association between urinary mercury concentration and allergic symptoms. As a result of multivariable logistic regression analyses, after adjusting for creatinine (Model 1), urinary mercury showed a positive correlation between atopic dermatitis in cycle 3 (OR [95% CI] = 1.36 [1.02, 1.81]) and cycle 4 OR ([95% CI] = 1.21 [0.64, 2.29]). After adjusting for all covariates in the 3rd and 4th cycles (Model 3), urinary mercury positively correlated with atopic dermatitis, asthma, and multimorbidity. Similar results were observed in pooled effects using two-cycle data, and significant positive correlations were found for atopic dermatitis and multimorbidity (Fig. 2).

**Table 2:** Association of urinary mercury level with current allergic symptoms.

Allergic symptoms	Crude model	Model 1^a^	Model 2^b^	Model 3^c^
	OR (95% CI)	OR (95% CI)	OR (95% CI)	OR (95% CI)
Cycle 3				
Atopic dermatitis	1.31 (0.99, 1.73)	1.36 (1.02, 1.81)	1.38 (1.02, 1.87)	1.38 (1.01, 1.89)
Asthma	1.41 (0.93, 2.13)	1.41 (0.57, 3.52)	1.48 (0.64, 3.44)	1.42 (0.60, 3.35)
Allergic rhinitis	0.94 (0.71, 1.23)	0.75 (0.54, 1.05)	0.77 (0.54, 1.10)	0.75 (0.52, 1.07)
Multimorbidity^1^	1.50 (1.09, 2.06)	1.34 (0.90, 1.99)	1.39 (0.92, 2.09)	1.38 (0.90, 2.13)
Cycle 4				
Atopic dermatitis	1.32 (0.78, 2.24)	1.21 (0.64, 2.29)	1.21 (0.64, 2.30)	1.20 (0.61, 2.37)
Asthma	0.40 (0.14, 1.13)	1.01 (0.13, 7.85)	1.44 (0.19, 11.13)	2.87 (0.49, 16.80)
Allergic rhinitis	1.20 (0.90, 1.60)	1.45 (1.00, 2.11)	1.45 (1.00, 2.10)	1.46 (1.01, 2.10)
Multimorbidity^1^	1.52 (0.87, 2.66)	1.61 (0.84, 3.10)	1.61 (0.84, 3.10)	1.74 (0.85, 3.52)

*CI* confidence interval, *OR* odds ratio.

^1^Multimorbidity was defined as the presence of two or more symptoms, and the statistics were calculated by comparing this group and the group with no symptoms.

Model 1 was adjusted for urinary creatinine level.

Model 2 was adjusted for all covariates in Model 1 and further adjusted for sex and age.

Model 3 was adjusted for all covariates in Model 2 and further adjusted for household income, parental education, BMI, and urinary cotinine level.

Table 3 shows the results of subgroup analyses to determine the relationship between urinary mercury concentration and allergic disease symptoms according to sex. For atopic dermatitis and multimorbidity, the pooled effects of the 3rd and 4th cycles showed a greater association in males than in female participants. The positive association between multimorbidity and mercury was significant only in boys.

**Table 3:** Association of urinary mercury level with current allergic symptoms according to sex.

Allergic symptoms	Sex	Cycle 3	Cycle 4	Pooled effects
		OR (95% CI)	OR (95% CI)	OR (95% CI)
Atopic dermatitis	Male	1.88 (1.01, 3.49)	1.24 (0.38, 4.13)	1.72 (0.99, 2.99)
	Female	1.13 (0.71, 1.81)	1.27 (0.57, 2.87)	1.16 (0.77, 1.74)
Asthma^1^	Male	0.79 (0.21, 3.03)	–	–
	Female	1.66 (0.77, 3.58)	5.95 (0.61, 58.55)	1.89 (0.92, 3.91)
Allergic rhinitis	Male	0.68 (0.45, 1.04)	1.71 (1.01, 2.87)	0.96 (0.69, 1.33)
	Female	0.80 (0.51, 1.26)	1.16 (0.68, 1.99)	0.94 (0.67, 1.32)
Multimorbidity^2^	Male	1.66 (0.77, 3.55)	2.36 (0.82, 6.79)	1.88 (1.01, 3.49)
	Female	1.24 (0.68, 2.26)	1.27 (0.40, 4.04)	1.25 (0.73, 2.14)

*CI* confidence interval, *OR* odds ratio.

^1^Statistics could not be calculated for empty cells because only rare cases of asthma were found in this sex subgroup.

^2^Multimorbidity was defined as the presence of two or more symptoms, and the statistics were calculated by comparing this group and the group with no symptoms.

All models were adjusted for urinary creatinine level, age, sex, urinary cotinine level, household income, parental education, and body mass index.

## Discussion

In this study, we investigated the association between urinary mercury concentrations and allergic diseases in children aged 6–11 years who participated in the KoNEHS cycles 3 and 4. After adjusting for all covariates, the urinary mercury level was positively associated with atopic dermatitis in 3rd cycle and with allergic rhinitis in 4th cycle. When we calculated the pooled effects using data from cycles 3 and 4, the OR for atopic dermatitis was 1.34 (95% CI = 1.01, 1.79), and the OR for allergic multimorbidity was 1.47 (95% CI = 1.01, 2.12). Additionally, the association with allergic multimorbidity was greater in boys than in girls.

The results of this study are consistent with the findings of previous studies on the effects of mercury exposure on allergic diseases. In studies from Korea, serum mercury levels were reported to have a positive correlation with the risk of atopic dermatitis in adults and infants^7,12^, and a significant positive correlation was observed with asthma in a longitudinal study of children^11^. However, a cross-sectional study in Germany and the United States reported no association with asthma^6,13^, which may be explained by the higher average body mercury levels in Korea compared to those in Germany and the United States^24^. The average mercury levels in children in this study were 0.40 µg/L for the 3rd and 4th cycles, which were higher than those in the United States NHANES (2015–2016) (0.25 µg/L for 3–11-year-olds) and Germany GerES V (2014–2017) (0.068 µg/L for 5–9-year-olds) in similar periods and age groups. Previous studies have attributed this difference in mercury levels to Korean eating habits, which include frequent consumption of grains and seafood^25,26^ and exposure to air pollution^27^. For these reasons, serum mercury levels are also high at 2.75 µg/L in adults based on the 3rd cycle of KoNEHS, which is similar to or lower than those in Japan and Taiwan, where similar diets are shared^28,29^.

Conversely, this study did not observe a consistent association with allergic rhinitis. Unlike atopic dermatitis and asthma, few studies have observed an association of allergic rhinitis with mercury, and no association was found in a study that examined overall allergic diseases^20^. Allergic rhinitis is classified into seasonal allergic rhinitis, affected by outdoor allergens such as pollen and perennial allergic rhinitis, affected by indoor allergens such as mold and animal hair. Allergic rhinitis is known to be affected by various environmental factors such as season, region, and lifestyle^30,31^. Moreover, in previous studies investigating the association between harmful environmental factors and allergic rhinitis, a significant negative association was observed for substances such as bisphenol S, vanadium, and 3,5,6-trichloro-2-pyridinol^32–34^. Considering these findings, the lack of consistent associations observed in this study may be due to differences in variables of the study environment that were not examined.

Various prior studies have reported on the biological mechanisms of low-level mercury exposure and changes in the immune system. Mercury exposure induces the proliferation of T and B lymphocytes by increasing the expression of major histocompatibility complex (MHC) class II molecules on antigen-presenting cells, and it increases serum immunoglobulin (Ig) G1 and IgE associated with allergic sensitization^9,35^. Mercury can also increase the levels of inflammatory cytokines such as interleukin-4 (IL-4), interleukin-13 (IL-13), and tumor necrosis factor-α (TNF-α) by stimulating T helper 2 (Th2) cells and worsen IgE-dependent diseases by interfering with the regulation of the immune system^8,36,37^. Sensitized Th2 cells can trigger allergic diseases when moving to the respiratory tract and various organs, and the atopic march may occur in which atopic dermatitis, asthma, and allergic rhinitis progress in sequence^38^. In this study, a significant positive association between mercury and multimorbidity was observed, and our results are supported by the fact that the association between each allergic disease has been frequently reported in several prior studies^39,40^.

Furthermore, this study showed a stronger association between mercury exposure and allergic diseases in boys, which is consistent with the findings of previous studies^11,41^. Although the mechanism by which allergic diseases have different effects by sex is not clearly understood, it is generally known that allergic diseases are more common in boys than in girls during infancy but more common in women after puberty^42^. This is thought to be because boys have a stronger allergic sensitization response than girls at a young age^43^. Moreover, boys are known to have slower lung function development than girls, so they are relatively vulnerable to the response of allergens^44^. After puberty, estrogen stimulates mast cells and eosinophils to promote allergic reactions, whereas androgens inhibit the differentiation of some T cell subsets, resulting in immunosuppressive effects^45–47^. Therefore, mercury exposure in boys appears to increase their susceptibility to allergic diseases, but follow-up studies are needed to confirm the results of this study.

This study has several limitations. First, since this study is a cross-sectional study, it is difficult to prove the causality of the result that urinary mercury concentration in children affects allergic disorders. Therefore, follow-up studies targeting children are necessary. Second, the evaluation of parameters such as allergic symptoms based on the parents' questionnaire responses may not reflect the actual disease status well. However, their responses are based on the diagnosis from experienced doctors, and information on allergic disorders of children based on parental surveys is relatively accurate^48^. In addition, information bias was minimized in this study by determining the presence of allergic disorders based on medication use and treatment. Third, due to the inherent limitations of using secondary data, data on long-term indicators such as hair mercury concentration and information about parental allergy history were not included in the analysis. However, the urinary mercury concentration is an indicator of chronic exposure to mercury^13^ and has been widely used as an indicator that can easily be obtained from a large number of samples^49^. In addition, even if some confounding variables were not considered, this study was based on KoNEHS, a large national bio-monitoring survey of Korean children. Therefore, our study provides sufficient statistical power to identify associations that may be less evident and thus provides reliable epidemiological evidence that mercury exposure in Korean children is associated with allergic diseases.

## Data Availability

The datasets analyzed during the current study are available on request at the National Institute of Environmental Research, Environmental Health Research Department, https://www.nier.go.kr/NIER/kor/op/nier-op-2001.do?menuNo=11000.

## References

[1] Boguniewicz M, Fonacier L, Leung DYM, O'Hehir RE, Holgate ST, Hershey GKK, Sheikh A (2022). Allergy Essentials.

[2] Ramírez-Marín HA, Silverberg JI (2022). Differences between pediatric and adult atopic dermatitis. Pediatr. Dermatol..

[3] Stern J, Pier J, Litonjua AA (2020). Asthma epidemiology and risk factors. Semin. Immunopathol..

[4] Oh I (2018). Association between particulate matter concentration and symptoms of atopic dermatitis in children living in an industrial urban area of South Korea. Environ. Res..

[5] Pacheco SE (2021). Climate change and global issues in allergy and immunology. J. Allergy Clin. Immunol..

[6] Wu KG, Chang CY, Yen CY, Lai CC (2019). Associations between environmental heavy metal exposure and childhood asthma: A population-based study. J. Microbiol. Immunol. Infect..

[7] Park H, Kim K (2011). Association of blood mercury concentrations with atopic dermatitis in adults: A population-based study in Korea. Environ. Res..

[8] Gardner RM, Nyland JF, Silbergeld EK (2010). Differential immunotoxic effects of inorganic and organic mercury species in vitro. Toxicol. Lett..

[9] Schiraldi M, Monestier M (2009). How can a chemical element elicit complex immunopathology? Lessons from mercury-induced autoimmunity. Trends Immunol..

[10] Dórea JG (2015). Exposure to mercury and aluminum in early life: Developmental vulnerability as a modifying factor in neurologic and immunologic effects. Int. J. Environ. Res. Public Health.

[11] Kim K-N, Bae S, Park HY, Kwon H-J, Hong Y-C (2015). Low-level mercury exposure and risk of asthma in school-age children. Epidemiology.

[12] Shin J (2019). The association between mercury exposure and atopic dermatitis in early childhood: A mothers and children's environmental health study. Epidemiology.

[13] Heinrich J, Guo F, Trepka MJ (2017). Brief report: Low-level mercury exposure and risk of asthma in school-age children. Epidemiology.

[14] Park S, Lee E-H, Kho Y (2016). The association of asthma, total IgE, and blood lead and cadmium levels. J. Allergy Clin. Immunol..

[15] Miyake Y, Tanaka K, Yasutake A, Sasaki S, Hirota Y (2011). Lack of association of mercury with risk of wheeze and eczema in Japanese children: The Osaka Maternal and Child Health Study. Environ. Res..

[16] Seo J-W, Hong Y-S, Kim B-G (2021). Assessment of lead and mercury exposure levels in the general population of Korea using integrated national biomonitoring data. Int. J. Environ. Res. Public Health.

[17] NIER (2018). Laboratory Procedures of Environmental Pollutants in Biospecimen.

[18] Esty B, Permaul P, DeLoreto K, Baxi SN, Phipatanakul W (2019). Asthma and allergies in the school environment. Clin. Rev. Allergy Immunol..

[19] Bousquet J (2015). Are allergic multimorbidities and IgE polysensitization associated with the persistence or re-occurrence of foetal type 2 signalling? The MeDALL hypothesis. Allergy.

[20] Koh HY (2019). Serum heavy metal levels are associated with asthma, allergic rhinitis, atopic dermatitis, allergic multimorbidity, and airflow obstruction. J. Allergy Clin. Immunol. Pract..

[21] Odebeatu CC, Taylor T, Fleming LE, Osborne NJ (2019). Phthalates and asthma in children and adults: US NHANES 2007–2012. Environ. Sci. Pollut. Res..

[22] Krebs NF (2007). Assessment of child and adolescent overweight and obesity. Pediatrics.

[23] Park S (2014). Environmental tobacco smoke exposure and children’s intelligence at 8–11 years of age. Environ. Health Perspect..

[24] Eom S-Y (2018). Lead, mercury, and cadmium exposure in the Korean general population. J. Korean Med. Sci..

[25] Lee JW (2012). Korea National Survey for environmental pollutants in the human body 2008: Heavy metals in the blood or urine of the Korean population. Int. J. Hyg. Environ. Health.

[26] Shin Y-Y, Ryu I-K, Park M-J, Kim S-H (2018). The association of total blood mercury levels and overweight among Korean adolescents: Analysis of the Korean National Health and Nutrition Examination Survey (KNHANES) 2010–2013. Korean J. Pediatr..

[27] Wang W, Wu F, Zheng J, Wong MH (2013). Risk assessments of PAHs and Hg exposure via settled house dust and street dust, linking with their correlations in human hair. J. Hazard. Mater..

[28] Eom S-Y (2014). Reference levels of blood mercury and association with metabolic syndrome in Korean adults. Int. Arch. Occup. Environ. Health.

[29] Tsai T-L (2019). Type 2 diabetes occurrence and mercury exposure—From the National Nutrition and Health Survey in Taiwan. Environ. Int..

[30] Brożek JL (2017). Allergic Rhinitis and its Impact on Asthma (ARIA) guidelines—2016 revision. J. Allergy Clin. Immunol..

[31] Meng Y, Wang C, Zhang L (2020). Advances and novel developments in allergic rhinitis. Allergy.

[32] Hwang M, Choi K, Park C (2022). Urinary levels of phthalate, bisphenol, and paraben and allergic outcomes in children: Korean National Environmental Health Survey 2015–2017. Sci. Total Environ..

[33] Ruan F (2022). Association between prenatal exposure to metal mixtures and early childhood allergic diseases. Environ. Res..

[34] Islam JY (2023). Respiratory and allergic outcomes among 5-year-old children exposed to pesticides. Thorax.

[35] Vas J, Monestier M (2008). Immunology of mercury. Ann. N. Y. Acad. Sci..

[36] de Vos G, Abotaga S, Liao Z, Jerschow E, Rosenstreich D (2007). Selective effect of mercury on Th2-type cytokine production in humans. Immunopharmacol. Immunotoxicol..

[37] Strenzke N (2001). Mercuric chloride enhances immunoglobulin E-dependent mediator release from human basophils. Toxicol. Appl. Pharmacol..

[38] Paller AS, Spergel JM, Mina-Osorio P, Irvine AD (2019). The atopic march and atopic multimorbidity: Many trajectories, many pathways. J. Allergy Clin. Immunol..

[39] Brzozowska A, Woicka-Kolejwa K, Jerzynska J, Majak P, Stelmach I (2022). Allergic rhinitis and house dust mite sensitization determine persistence of asthma in children. Indian J. Pediatr..

[40] Sigurdardottir ST (2021). Prevalence and early-life risk factors of school-age allergic multimorbidity: The EuroPrevall-iFAAM birth cohort. Allergy.

[41] Carrasco P (2021). Pre and postnatal exposure to mercury and respiratory health in preschool children from the Spanish INMA Birth Cohort Study. Sci. Total Environ..

[42] Shah R, Newcomb DC (2018). Sex bias in asthma prevalence and pathogenesis. Front. Immunol..

[43] Leffler J, Stumbles PA, Strickland DH (2018). Immunological processes driving IgE sensitisation and disease development in males and females. Int. J. Mol. Sci..

[44] Mogensen I (2022). Lung function in young adulthood: Differences between males and females with asthma. ERJ Open Res..

[45] Foo YZ, Nakagawa S, Rhodes G, Simmons LW (2017). The effects of sex hormones on immune function: A meta-analysis. Biol. Rev..

[46] Kanda N, Hoashi T, Saeki H (2019). The roles of sex hormones in the course of atopic dermatitis. Int. J. Mol. Sci..

[47] Keselman A, Heller N (2015). Estrogen signaling modulates allergic inflammation and contributes to sex differences in asthma. Front. Immunol..

[48] Strömberg Celind F, Vasileiadou S, Goksör E (2021). Parental questionnaires provided reliable data on childhood asthma compared with national registers. Pediatr. Allergy Immunol..

[49] He L (2021). Temporal trends of urinary mercury in Chinese people from 1970s to 2010s: A review. Ecotoxicol. Environ. Saf..

